# Interplay between TRIM7 and antiviral immunity

**DOI:** 10.3389/fcimb.2023.1256882

**Published:** 2023-08-31

**Authors:** Yiyang Liu, Lu Jiang, Xuemeng Sun, Yixuan Song, Yihan Liu, Leiliang Zhang

**Affiliations:** ^1^ Department of Clinical Laboratory Medicine, The First Affiliated Hospital of Shandong First Medical University & Shandong Provincial Qianfoshan Hospital, Jinan, Shandong, China; ^2^ Medical Science and Technology Innovation Center, Shandong First Medical University & Shandong Academy of Medical Sciences, Jinan, Shandong, China; ^3^ Department of Pathogen Biology, School of Clinical and Basic Medical Sciences, Shandong First Medical University & Shandong Academy of Medical Sciences, Jinan, Shandong, China

**Keywords:** TRIM7, E3 ubiquitin ligase, MAVS, STING, enterovirus

## Abstract

TRIM7 has been demonstrated to have significant roles in promoting host defense against viral infections and regulating immune signaling pathways. As an E3 ubiquitin ligase, it catalyzes the ubiquitination of various substrates, including adaptor proteins (MAVS and STING) and transcription factors (NF-κB and IRF3), thereby exerting positive or negative regulation on immune signal pathways. However, viruses have developed immune evasion mechanisms to counteract TRIM7. Some viruses can inhibit TRIM7 function by targeting it for degradation or sequestering it away from its targets. Moreover, TRIM7 may even facilitate viral infection by ubiquitinating viral proteins, including envelope proteins that are critical for tissue and species tropism. A comprehensive understanding of the interaction between TRIM7 and antiviral immunity is crucial for the development of innovative treatments for viral diseases.

## 1 Introduction

The tripartite motif (TRIM) family is a highly conserved and dynamically evolving protein family that plays crucial roles in various cellular processes, such as signal transduction, tumorigenesis, innate immunity, and antiviral immunity (Hatakeyama, 2017; Koepke et al., 2021). TRIM proteins are characterized by the presence of the conserved RING finger-B box-Coiled coil (RBCC) domain architecture, which includes a RING domain, one or two B-boxes, and coiled-coil domains, along with a variable C-terminal domain. The RING domain facilitates the recruitment of ubiquitin-loaded E2 enzymes and catalyzes the transfer of ubiquitin to the substrate, while the B-box and coiled-coil domains play regulatory roles in oligomerization and ligase activity. The C-terminal domain (CTD), on the other hand, typically confers substrate specificity to the TRIM protein. Different types of C-terminal domains have been identified in TRIM proteins(Koepke et al., 2021). One such example is the PRY-SPRY domain, which serves as a protein-protein interaction module commonly found in eukaryotic proteins. It features a twisted β-sandwich fold composed of two β-sheets and several loops of varying lengths (Koepke et al., 2021). Notably, the PRY-SPRY domain has been observed exclusively in vertebrates (Liang et al., 2022).

**Figure 1:**
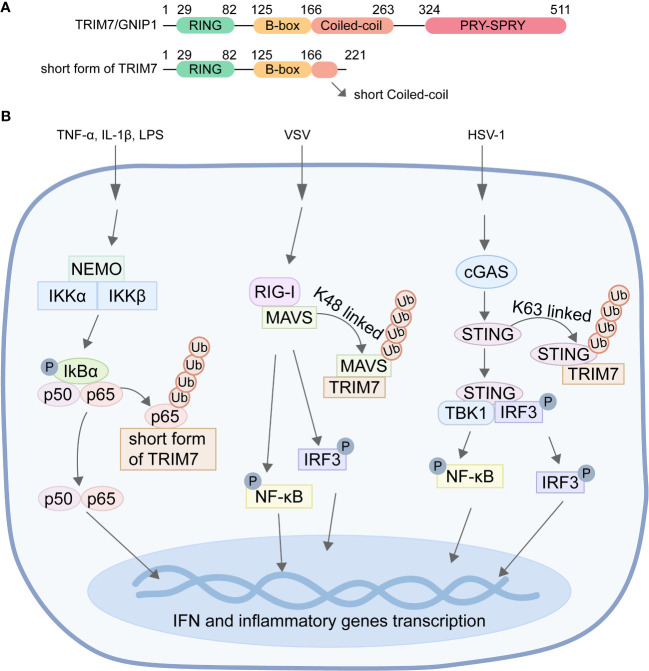
The ubiquitination of NF-κB, MAVS, and STING mediated by TRIM7. **(A)** Diagram of the domains of TRIM7/GNIP1 and short form of TRIM7. **(B)** TRIM7 facilitates the K48-linked ubiquitination of p65, resulting in the degradation of p65 through the proteasome pathway, ultimately leading to the inhibition of NF-κB signal pathway transduction. Moreover, TRIM7 promotes K48-linked ubiquitination of MAVS and STING, thereby facilitating their degradation and subsequently inhibiting the transcription of IFN and inflammatory cytokine genes.

TRIM7, encoded by the trim7 gene, is the seventh member of the TRIM family. It exists in four isoforms, namely TRIM7/GNIP1, GNIP2, GNIP3, and short form of TRIM7 (Zhai et al., 2004). Among these isoforms, only TRIM7/GNIP1 and short form of TRIM7 possess the characteristic RING domain found in the TRIM family. Both GNIP1 and TRIM7 also feature B-box and coiled-coil domains, in addition to a CTD (**Figure 1A**). However, GNIP1 contains the PRY-SPRY domain in its CTD, which is implicated in antiviral responses and innate immune pathways. In contrast, the CTD of short form of TRIM7 is considerably shorter, consisting of only 15 amino acids (**Figure 1A**). This domain plays a crucial role in inhibiting tumor cell proliferation and migration, while promoting apoptosis. Overall, the distinct C-terminal domains present in TRIM7/GNIP1 and short form of TRIM7 contribute to their specific functions in various cellular processes.

TRIM7 is characterized by the conserved RBCC domain architecture, along with a CTD (**Figure 1A**). The RING domain comprises conserved cysteine and histidine residues that bind to two zinc atoms in a cross-brace arrangement. It is essential for recruiting ubiquitin-loaded E2 and catalyzing the conjugation of ubiquitin to substrates. The CC domain, in combination with the B-box domain, has been proposed as a mediator of protein-protein interactions, particularly homomeric and heteromeric interactions. The CTD of TRIM7 consists of the PRY and SPRY domains, either combined as PRY-SPRY or individually (Liang et al., 2022; Ru et al., 2022). The PRY-SPRY domain, a protein-protein interaction module found widely in eukaryotic proteins, features a twisted β-sandwich fold composed of two β-sheets. The PRY-SPRY domain of TRIM7 possesses a unique binding site that promiscuously interacts with proteins containing a C-terminus ending in a helix-ΦQ motif (Liang et al., 2022; Ru et al., 2022). The reason why TRIM7 has been reported to bind glycogenin-1 (GYG1), RACO-1, and Coxsackie virus B3 (CVB3) 2BC is due to the presence of this structural signature in these proteins.

The ubiquitin-proteasome system (UPS) is responsible for the targeted degradation of proteins within cells and plays a crucial role in regulating various cellular processes, including viral replication and host immune signaling pathways (Pla-Prats and Thomä, 2022). This system involves a cascade of enzymes, including the E1 (ubiquitin activating enzyme), E2 (ubiquitin binding enzyme), and E3 (ubiquitin protein ligase) enzymes, which collaborate to attach ubiquitin molecules to target proteins. Ubiquitin has seven lysine residues and one methionine residue. Ubiquitination primarily occurs through the linkage of lysine residues and methionine residue (Kolla et al., 2022). The K48 linkage is frequently linked to targeting proteins for degradation by the proteasome (Raja and Sen, 2022), while the K63 linkage typically regulates signaling pathways without involving protein degradation (Madiraju et al., 2022). Although K48 and K63 linkages are more common and extensively studied in ubiquitination, methionine linkage also plays a role in regulating protein function and fate in specific circumstances. Research on methionine linkage is still ongoing to uncover its specific role and mechanisms in cellular regulation. The UPS system serves a vital role in regulating viral replication and modulating host immune signal pathways. By selectively targeting proteins for degradation or modifying their function through ubiquitination, the UPS system prevents tissue damage resulting from excessive activation of these processes. Dysregulation of the UPS system has been implicated in the development of numerous human diseases, including cancer, neurodegenerative disorders, and inflammatory diseases (Pohl and Dikic, 2019).

In recent years, numerous studies have demonstrated that TRIM7 exhibits the ability to catalyze the ubiquitination of diverse substrates, which has been associated with its antiviral and immune regulatory functions (**Table 1**). In this review, we aim to provide a comprehensive summary of these findings and emphasize the intricate role of TRIM7 in virus infection and innate immunity.

**Table 1:** The involvement of TRIM7 in the viral life cycle and innate immunity.

Function	Function	References
Antiviral role of TRIM7	TRIM7 exhibits inhibitory effects against various human enteroviruses including type B, EV-A71, EV-D68, Echovirus11, PV, and NoVs. Additionally, TRIM7 also demonstrates inhibitory activity against non-human primate enteroviruses such as EV-B112, EV-B113, and EV-B114.	(Fan et al., 2021; Liang et al., 2022; Luptak et al., 2022)
Proviral role of TRIM7	TRIM7 mediates K63-linked ubiquitination of the ZIKV envelope protein, playing a critical role in determining the tissue tropism of ZIKV replication *in vivo*.	(Giraldo et al., 2020)
**Enterovirus evasion strategies for antiviral role of TRIM7**	TRIM7 undergoes cleavage by enterovirus 3C protease (3Cpro), resulting in the generation of a C-terminal cleavage fragment. This cleavage fragment inhibits the activity of the E3 ligase, thereby impairing the antiviral activity of TRIM7.	(Fan et al., 2022)
Enhance of TLR4 signaling pathway by TRIM7	TRIM7 plays a role in the regulation of innate immunity through the TLR4 signaling pathway and acts as a positive regulator of MAPKs, NF-κB, and IRF-3 signal pathways that are associated with TLR4.	(Lu et al., 2019)
Enhance of IFN-β signaling pathway by TRIM7	TRIM7 enhances the IFN-β signaling pathway to suppress EMCV replication.	(Li et al., 2023)
Ubiquitination of NF-κB by short form of TRIM7	Short form of TRIM7 interacts with p65 and facilitates the ubiquitination of p65, leading to its accelerated degradation *via* the proteasome pathway. As a result, this process ultimately inhibits the transduction of the NF-κB signal pathway.	(Jin et al., 2020)
Ubiquitination of MAVS by TRIM7	TRIM7 induces K48-linked ubiquitination of MAVS, leading to its subsequent degradation through the proteasome. This negative regulation of MAVS by TRIM7 attenuates the antiviral immune response.	(Yang et al., 2021)
Ubiquitination of STING by TRIM7	TRIM7 promotes K48-linked ubiquitination modification of STING, resulting in its degradation and subsequent inhibition of the cGAS/STING-mediated signaling pathway. Furthermore, TRIM7 has been identified as a negative regulator of STING expression.	(Yang et al., 2020)

## 2 Direct role for TRIM7 in the life cycle of viruses

### 2.1 Antiviral role of TRIM7

TRIM7 has been demonstrated to restrict enterovirus replication by recognizing the C-terminal domain of the nonstructural protein 2C of CVB3 *via* its PRY-SPRY domain (Fan et al., 2021). This recognition leads to the ubiquitination and subsequent degradation of the virus 2BC protein, ultimately inhibiting enterovirus replication. The crystal structure of the TRIM7-2C complex has been elucidated, revealing that the PRY-SPRY domain of TRIM7 specifically recognizes the C-terminal Gln329 of 2C, while other residues of TRIM7 interact with Phe328 and Leu327 (Ru et al., 2022). TRIM7 modifies the enterovirus 2BC protein through K48-linked ubiquitination, resulting in its degradation by the proteasome and exerting a direct antiviral effect. However, it is noteworthy that CVB3 variants resistant to TRIM7 can emerge through mutations in the 2C protein, such as the T323A mutation, which enables CVB3 to evade TRIM7-mediated inhibition (Fan et al., 2021).

TRIM7 has also been found to recognize and interact with proteins or peptides containing the C-terminal helix-ΦQ motif, which includes proteins ending with either “LQ” or “FQ” (Luptak et al., 2022). This recognition mechanism is based on the observation that many viral genomes encode their major replicase components as single polypeptides that are subsequently processed into individual proteins by viral 3C proteases, which cleave after Q residues. TRIM7 has been demonstrated to inhibit the replication of various human enteroviruses, including EV-A71, EV-D68, Echovirus11, PV, and NoVs, as well as non-human primate enteroviruses EV-B112, EV-B113, and EV-B114, by recognizing and targeting their C-terminal helix-ΦQ motif-containing proteins(Fan et al., 2021; Luptak et al., 2022). However, TRIM7 does not exhibit inhibitory effects on other viruses, such as mouse enterovirus (MenV), yellow fever virus (YFV), Zika virus (ZIKV), and respiratory syncytial virus (RSV).

While TRIM7 has been found to interact with proteins containing the C-terminal helix-ΦQ motif, it is important to note that not all proteins ending with “LQ” or “FQ” are necessarily substrates of TRIM7. Other factors, such as spatial accessibility, may also influence the interaction. In the case of SARS-CoV-2, which possesses 10 proteins ending with “LQ” or “FQ”, some studies suggest that TRIM7 can bind and degrade certain SARS-CoV-2 proteins, including NSP5 and NSP8. However, the restriction of SARS-CoV-2 replication by TRIM7 has not been demonstrated(Liang et al., 2022). It is possible that the restriction of SARS-CoV-2 by TRIM7 may require a cell line that is more physiologically relevant to SARS-CoV-2 or expresses higher levels of TRIM7. Additionally, it is possible that TRIM7 may not interact with SARS-CoV-2 during live virus infection or that the degradation of SARS-CoV-2 proteins by TRIM7 may not sufficiently impact virus replication. It is also plausible that SARS-CoV-2 may antagonize the function of TRIM7. Overall, while TRIM7 has shown antiviral effects against certain human enteroviruses, its role in the context of SARS-CoV-2 infection remains incompletely understood and requires further investigation.

### 2.2 Proviral role of TRIM7

ZIKV, a member of the *Flaviviridae* family, is known to cause congenital neurological diseases, such as microcephaly (Giraldo et al., 2023). The envelope protein (E) of ZIKV plays a crucial role in viral entry into host cells by facilitating virus adhesion and inducing viral endosomal membrane fusion. TRIM7 mRNA can be detected in sites known for ZIKV replication, such as the placenta, brain, and testiss. Knocking down TRIM7 expression using siRNA significantly reduces ZIKV replication in placental JEG-3 and brain-derived HTB-15 cells. These pro-viral effects of TRIM7 depend on the presence of an intact K38 residue on the E protein (Giraldo et al., 2020). While ZIKV E-WT replicates at lower levels in TRIM7 JEG-3 CRISPR Knockout (TRIM7 KO) cells, no additional difference is observed when infecting with the ZIKV E-K38R mutant (a recombinant virus lacking ubiquitination at E-K38 during infection) compared to WT and TRIM7 KO cells. Co-immunoprecipitation experiments confirm the interaction between endogenous TRIM7 and E in ZIKV-infected cells. Additionally, TRIM7, along with the E2-conjugating enzyme UbcH5a, previously identified as interacting and facilitating TRIM7-mediated K63-linked ubiquitination, directly ubiquitinates recombinant ZIKV-E at both K38 and K281 in an *in vitro* ubiquitination assay (Giraldo et al., 2020).

TRIM7 plays a significant role in determining the tissue tropism of ZIKV replication *in vivo* (Giraldo et al., 2020). The virus titer in serum, kidney, eyes, brain, and reproductive tissues (uterus and testis) of Trim7−/− mice was significantly lower compared to wild-type mice, while no significant differences were observed in the heart, liver, lungs, and muscles. ZIKV virus is released from infected cells after being ubiquitinated by TRIM7 (Giraldo et al., 2020). An anti-K63-Ub antibody could neutralize ZIKV replication in cells and *in vivo* (Giraldo et al., 2020). These findings imply that TRIM7 could be a potential target for the development of antiviral strategies against ZIKV infection.

### 2.3 Evasion strategies for antiviral role of TRIM7

TRIM7 exhibits antiviral activity against enteroviruses by targeting the viral 2BC protein for ubiquitination and subsequent proteasome-dependent degradation. However, enteroviruses can also counteract the antiviral effects of TRIM7 through various mechanisms. For instance, CVB3 and PV can cleave TRIM7 using their 3C protease (3Cpro), while EVA71 does not possess this ability. The cleavage of TRIM7 by CVB3 and PV 3Cpro occurs at Q24, which corresponds to a conserved region recognized by 3Cpro. The resulting cleavage fragment of TRIM7, which retains classical TRIM domains such as RING and PRY-SPRY, inhibits the E3 ligase activity of TRIM7, thereby impairing its antiviral function (Fan et al., 2022). Interestingly, the Q24 cleavage site is highly conserved in mammals, with the exception of four marsupials that have histidine 24 (H24) residues. The natural substitution of glutamate with histidine (Q24H) in koala TRIM7 renders the N-terminal resistant to CVB3 3Cpro cleavage. However, the insertion of 27 amino acids in the PRY-SPRY domain of koala TRIM7 introduces another cleavage site at Q338 (Fan et al., 2022). This suggests that certain enteroviruses may have evolved to target marsupial TRIM7, even in the absence of the canonical Q24. Hence, TRIM7 may serve as a target for enterovirus 3Cpro as a potential evasion strategy, and mammalian TRIM7 proteins may possess distinct antiviral properties against different enteroviruses.

## 3 The role for TRIM7 in innate immunity

### 3.1 The role for TRIM7 in TLR4 mediated signaling transduction

When microorganisms infect the host, it triggers local or systemic inflammatory responses, and the initial recognition of infectious substances is mediated by cell pattern recognition receptors (PRRs). Among these receptors, TLR4 is extensively studied as a pattern recognition receptor for bacterial lipopolysaccharide (LPS), playing a crucial role in the host’s recognition and elimination of invasive pathogens (Zhang et al., 2022). As a signal transduction PRR, TLR4 directly recognizes and binds to LPS, leading to the activation of macrophages and subsequent activation of IRF and nuclear factor κB (NF-κB) signaling pathways. This activation induces the production of type I interferon (IFN-α/β) and proinflammatory cytokines such as IL-1. The activation of immune responses is vital for the host to mount an effective defense against invading pathogens.

Recent studies have demonstrated that TRIM7 overexpression significantly enhances the LPS-induced expression of TNF-α, IL-6, and IFN-β in macrophages (Lu et al., 2019). Conversely, knockdown of TRIM7 inhibits the production of these pro-inflammatory cytokines. Further investigations have revealed that TRIM7 is involved in the regulation of innate immunity through the TLR4 signaling pathway, and it positively regulates MAPKs, NF-κB, and IRF-3 signal pathways, with the RING domain of TRIM7 playing a major role. By modulating TRIM7 expression, it is possible to reduce the production of inflammatory cytokines, thus holding potential therapeutic value for treating infectious diseases.

### 3.2 The role for TRIM7 in IFN-β mediated signaling transduction

IFN serves as the host’s primary defense against viruses, triggering the expression of numerous interferon-stimulated genes (ISGs) that actively combat viral infections. Encephalomyocarditis virus (EMCV) is a zoonotic virus belonging to the *Picornaviridae* family, capable of infecting a wide range of mammals and rodents. EMCV infections can lead to various symptoms and diseases, including myocarditis (inflammation of the heart muscle), encephalitis (inflammation of the brain), neurological disorders, reproductive diseases, and diabetes (Carocci and Bakkali-Kassimi, 2012). While human infections with EMCV are rare, they can occur through exposure to infected animals or consumption of contaminated food products.

A recent study demonstrated that the overexpression of TRIM7 in HEK293T cells significantly reduced EMCV replication, indicating the antiviral activity of TRIM7 against EMCV (Li et al., 2023). Additionally, this study revealed that TRIM7 upregulated the mRNA levels of IFN-β and several other ISGs, including ISG15, ISG56, and OAS1. Silencing TRIM7 expression using siRNA in HEK293T cells enhanced EMCV replication and disrupted IFN-β promoter activity (Li et al., 2023). These findings provide valuable insights into the mechanisms underlying the antiviral effects of TRIM7 against EMCV and suggest that TRIM7 could serve as a promising target for the development of novel antiviral therapies.

### 3.3 Ubiquitination of NF-κB by short form of TRIM7

The NF-κB/Rel protein family is an inducible transcription factor that plays a vital role in various biological processes, including cell proliferation, immune response, and tumorigenesis (Capece et al., 2022). NF-κB primarily functions as a heterodimer complex composed of p65/RelA and p50, and its activation typically involves IκB degradation. However, emerging evidence suggests that the degradation of p65 protein is crucial for terminating NF-κB signaling. Several factors, including the ubiquitin-proteasome system, autophagy, and caspases, regulate the degradation of p65, which is mediated by the proteasome. Various stimuli, such as TNF-α, IL-1β, and LPS, can induce p65 degradation, thereby negatively regulating NF-κB signaling. Consequently, p65 degradation serves as an important regulatory mechanism for terminating NF-κB signaling and maintaining cellular homeostasis.

Studies have shown that short form of TRIM7 exhibits ubiquitin ligase activity on p65 (Jin et al., 2020). Short form of TRIM7 interacts with p65 and promotes the ubiquitination of p65, leading to its accelerated degradation through the ubiquitin-proteasome system. This ultimately inhibits the transduction of the NF-κB signaling pathway (**Figure 1B**). Although the interaction between TRIM7/GNIP1 and p65 has also been observed, TRIM7/GNIP1 does not promote the degradation and ubiquitination of p65. Therefore, different regions of the C-terminal of the E3 ligase are crucial for the interaction with p65. Short form of TRIM7 regulates the NF-κB pathway and affects downstream targets through its ubiquitin ligase activity, while TRIM7/GNIP1 may impact the NF-κB pathway in a manner independent of the ubiquitin-proteasome system by interacting with p65 in different regions than TRIM7. These findings unveil a novel negative regulatory mechanism of the NF-κB signaling pathway involving short form of TRIM7 and confirm the repressor function of short form of TRIM7 in innate immunity (Jin et al., 2020).

### 3.4 Ubiquitination of MAVS by TRIM7

During virus infection and replication, viral nucleic acids bind to PRRs in host cells, triggering a series of signal cascade reactions that induce the expression of cytokines and chemokines such as IFN and TNF. For RNA viruses, PRRs such as TLR and RIG-I recognize invading pathogens and transmit signals to MAVS. MAVS, in turn, activates signal pathways including NF-κB and IRF3 by stimulating downstream TBKI complex and IKK complex, thereby further activating IFN-α/β expression and inducing a natural antiviral immune response in cells (Ren et al., 2020). Therefore, MAVS plays a crucial role in innate immunity and serve s as the central regulator of the anti-RNA virus response. The activity and stability of MAVS are tightly controlled through ubiquitination and deubiquitination.

Recent studies have revealed that TRIM7 functions as a negative regulator of RIG-I-mediated signaling pathways (Yang et al., 2021). During vesicular stomatitis virus (VSV) infection, overexpression of TRIM7 promotes K48-linked ubiquitination of MAVS, leading to its subsequent proteasome-dependent degradation in a dose-dependent manner (**Figure 1B**). Compared to wild-type mice, TRIM7 KO mice exhibited significantly increased levels of MAVS protein in tissues and cells, as well as elevated levels of cytokines such as type I interferon in serum (Yang et al., 2021). These findings indicate that TRIM7 deficiency enhances the innate immune response stimulated by RNA viruses and confers protection against RNA virus infection in mice. In summary, TRIM7 regulates the host cell’s antiviral immune response against RNA virus invasion by modifying MAVS with ubiquitin to promote its degradation.

### 3.5 Ubiquitination of STING by TRIM7

When DNA viruses infect host cells, they are recognized by the cytoplasmic pattern recognition receptor cGAS. cGAS catalyzes the synthesis of cGAMP from cytoplasmic ATP and GTP, acting as a second messenger. This cGAMP molecule then activates the transmembrane protein STING, which is located on the endoplasmic reticulum. Activation of STING leads to the production of IFN and subsequent initiation of the innate immune responses (Hopfner and Hornung, 2020). Mice deficient in STING are susceptible to fatal infections caused by DNA viruses such as HSV-1 and vaccinia virus, and display impaired production of type I interferon in response to cytoplasmic DNA. Recent studies have demonstrated that TRIM7 accumulates on the endoplasmic reticulum upon HSV-1 infection, with its PRY-SPRY domain responsible for interacting with STING. TRIM7 induces K48-linked ubiquitination modification of STING, resulting in a dose-dependent inhibition of STING protein expression. This negative regulation by TRIM7 suppresses the cGAS/STING-mediated signal transduction, thereby inhibiting STING-induced IFN-β activation (**Figure 1B**). Furthermore, TRIM7 deficiency enhances the innate immune response triggered by exogenous cytoplasmic DNA in bone marrow-derived dendritic cells (BMDCs) and provides protection against DNA virus infection in mice (Yang et al., 2020).

## 4 Conclusion and prospect

This review provides insights into the molecular mechanism by which the E3 ubiquitin ligase TRIM7 regulates RNA and DNA virus infections through ubiquitination (**Table 1**). These findings enhance our understanding of the innate immune response mechanisms against viruses and lay a theoretical foundation for the future development of antiviral drugs.

TRIM7 is an E3-Ub ligase that can either promote or inhibit virus infection (**Table 1**). For instance, enteroviruses such as CVB3 have developed multiple mechanisms to counteract the antiviral effects of TRIM7, thereby enhancing their replication (Fan et al., 2022). In the case of ZIKV, the E protein undergoes K63-linked polyubiquitination catalyzed by TRIM7, resulting in a permissive environment for virus replication in specific target tissues (Giraldo et al., 2020). This promotes enhanced replication in the brain and reproductive tissues, leading to increased pathogenesis *in vivo*. Furthermore, TRIM7 was identified through CRISPR screening as a host factor that inhibits norovirus replication in mice, indicating its broader role in antiviral immunity (Orchard et al., 2019). Additionally, during EMCV infection, TRIM7 actively participates in the IFN-β signaling pathway (Li et al., 2023). It interacts with MAVS and modulates the activity of the IFN-β promoter triggered by EMCV, ultimately inhibiting EMCV replication and its associated effects. TRIM7 exhibits both positive and negative regulatory functions in the IFN signaling pathway. Researchers speculate that the specific type of virus, infective doses, and duration of infection may contribute to the observed differences in TRIM7’s effects.

In addition, examining phylogeny and tissue expression provides valuable insights into the role of TRIM7. The evolutionary age of TRIM7 aligns reasonably well with the emergence of the helix-ΦQ motif in glycogenin (Malfavon-Borja et al., 2013). Tissue-specific expression is another relevant factor, and in this regard, TRIM7 and glycogenin demonstrate similarity. TRIM7 is primarily expressed in skeletal muscle and the brain, which are also regions with high glycogenin expression (Montori-Grau et al., 2018). Additionally, the Trim7 gene lacks an interferon-stimulated response element (ISRE) or gamma interferon-activated site (GAS), and its expression remains unaffected by interferon stimulation (Carthagena et al., 2009). Collectively, these findings do not strongly support TRIM7 as a broad-spectrum antiviral restriction factor. Its involvement in innate immunity appears to be limited.

Future investigations should focus on exploring TRIM7’s expression in naturally occurring tissues and utilizing *in vivo* animal models to further investigate its antiviral and innate immune effects. This research will aid in distinguishing TRIM7’s antiviral and proviral effects, facilitate the design of new antiviral strategies to combat viral immune evasion and the enhanced pathogenicity of mutant strains, and ultimately clarify whether pharmacological approaches can be employed to regulate TRIM7 activity for the treatment of related diseases. These efforts will provide a solid foundation for the future development of antiviral drugs.

## Author contributions

YiyL: Writing – original draft. LJ: Writing – review & editing. XS: Writing – review & editing. YS: Writing – review & editing. YihL: Software, Writing – review & editing. LZ: Conceptualization, Writing – review & editing.
